# Epithelial thickness gradients as a diagnostic biomarker for early-stage keratoconus

**DOI:** 10.1007/s10792-026-04091-x

**Published:** 2026-04-29

**Authors:** Zane Jansone-Langina, Jana Gertnere, Viktorija Kezika, Carlos Rocha-de-Lossada, José-María Sánchez-González

**Affiliations:** 1 Department of Optometry and Vision Science, University of Latvia, Riga, Latvia; 2 Dr. Solomatin Eye Center, Riga, Latvia; 3 Qvision, Ophthalmology Department, Vithas Almeria Hospital, 04120 Almeria, Spain; 4 Ophthalmology Department, Vithas Malaga, 29016 Malaga, Spain; 5 Hospital Civil Square, Regional University Hospital of Malaga, 29009 Malaga, Spain; 6 Department of Surgery, Ophthalmology Area, University of Seville, 41009 Seville, Spain; 7 Departament of Physics of Condensed Matter, Optics Area, Pharmacy Faculty, University of Seville, Seville, Spain

**Keywords:** Keratoconus, Epithelial thickness, Diagnostic biomarker, Early diagnosis

## Abstract

**Purpose:**

To evaluate peripheral-central epithelial thickness (ET) gradients in eyes with keratoconus and regular astigmatism, and to determine their potential utility as a non-invasive biomarker for disease detection in early-stage keratoconus.

**Methods:**

This retrospective cross-sectional analyzed ET with spectral-domain OCT at the cone apex and at 3 mm in the superior, temporal, nasal, and inferior directions. Epithelial thickness gradients (ΔET) were calculated as the difference between peripheral and central ET. Statistical comparisons were performed using non-parametric tests, with significance set at *p* < 0.05.

**Results:**

A total of 396 eyes (208 keratoconus, 188 regular astigmatism) were analyzed. Central ET was significantly reduced in KC (38.55 ± 5.42 µm) vs controls (48.78 ± 3.94 µm; *p* < 0.001). While peripheral thickness showed minor differences, the ET gradients (ΔET) were markedly steeper in KC across all quadrants (temporal, superior, nasal, inferior, *p* < 0.001). Within Stage I KC, eyes with more advanced disease exhibited steeper ΔET values, particularly in the nasal and temporal directions. Mean ΔET, demonstrated significant differences between KC (8.43 ± 4.97 µm) and controls (− 1.55 ± 1.77 µm;* p* < 0.001), and correlated positively with steep keratometry (r = 0.521, *p* < 0.001) and negatively with thinnest corneal thickness (TCT) (r =  − 0.641, *p* < 0.001).

**Conclusion:**

ΔET is markedly steeper in keratoconus than in regular astigmatism—even in Stage I—and correlates with steep keratometry and TCT. With areas under the curves (AUCs) up to 0.976, ΔET provides a rapid, non-invasive epithelial-based biomarker for early detection and staging of keratoconus. Its utility for monitoring progression remains to be established in prospective longitudinal studies.

*What is known*

- In keratoconus, the corneal epithelium exhibits central thinning surrounded by relative peripheral thickening, which can mask or precede tomographic abnormalities.
- Spatial mapping of epithelial thickness provides valuable information for detecting and characterizing corneal ectasia.

*What is new*

- The peripheral–central epithelial thickness gradient is consistently steeper in keratoconus compared with regular astigmatism, even in the earliest disease stages.
- This gradient demonstrates strong discriminatory ability between keratoconus and non-ectatic corneas, supporting its use as an adjunct diagnostic parameter.
- The gradient reflects disease severity in this cross-sectional cohort and may support staging; its value for monitoring progression requires longitudinal validation.

## Introduction

Keratoconus (KC) is a progressive, non-inflammatory ectatic disorder with inflammatory components of the cornea characterized by stromal thinning and conical protrusion, leading to irregular astigmatism and visual distortion [1–3]. The disease typically manifests during adolescence or early adulthood, with a bilateral but often asymmetric presentation[3, 4]. Its progression can result in significant visual impairment, making early detection a critical objective in clinical practice [5].

Several classification systems have been developed to stratify KC severity and guide management. The Amsler-Krumeich classification is based primarily on anterior corneal curvature and central corneal thickness (CCT) [2], while the more recent Belin ABCD system incorporates posterior corneal curvature and thinnest corneal thickness (TCT), improving early detection [6]. The ABCD classification includes anterior (A) and posterior (B) corneal curvature, thinnest corneal thickness (C), and distance visual acuity (D), all measured at the thinnest point of the cornea rather than the apex, as this better represents the location of the cone [7]. In contrast, the Belin/Ambrosio Enhanced Ectasia Display (BAD), introduced in 2008, is based on elevation data from anterior and posterior corneal surfaces relative to a best-fit sphere (BFS), and has evolved in its latest versions (e.g., BAD-D v4) to improve ectasia susceptibility detection [8].

Ambrosio et al. introduced the Corvis Biomechanical Index (CBI) with a 0.5 cutoff to distinguish keratoconus from healthy corneas [9, 10], and later demonstrated its utility in detecting biomechanical abnormalities even in eyes with normal topography and tomography. Ambrosio et al. reported a TBI cutoff of 0.29 (area under the curve, AUC = 0.985) for identifying ectasia in very asymmetric eyes with normal fellow eye topography, and later updated TBI-2 achieved an AUC of 0.945 with a cutoff of 0.43 [11]. However, these methods may overlook epithelial remodeling, a key adaptive process that can mask underlying stromal abnormalities, especially in early-stage disease [12].

The corneal epithelium plays a compensatory role in KC, smoothing stromal surface irregularities to preserve optical quality. Optical coherence tomography (OCT) studies have demonstrated epithelial thinning over the cone apex and relative thickening in surrounding areas, creating the so-called “doughnut pattern” [12–16]. This remodeling may obscure tomographic signs of ectasia in early stages, reducing the sensitivity of topography-based screening tools. Importantly, epithelial thickness gradients (∆ET), rather than absolute values, may better reflect the biomechanical and structural changes occurring in the cornea [17, 18].

In this context, the analysis of ∆ET emerges as a promising avenue. These gradients may capture spatial variations in epithelial response to stromal deformation, potentially distinguishing keratoconus from regular astigmatism, where such patterns are not typically present [19, 20]. As such, epithelial mapping could serve as a non-invasive biomarker for early disease detection and staging, helping to guide clinical decision-making and the timing of therapeutic interventions. Its value for monitoring progression remains hypothetical until validated prospectively. [21, 22].

The aim of this study is to assess whether peripheral-central ∆ET differ between KC and regular astigmatism, and to explore their potential as diagnostic biomarker of disease in patients with Stage I keratoconus.

## Method

### Design

This study was designed as a retrospective cross-sectional observational analysis based on anonymized clinical records. Data were collected between January 2023 and December 2024 from patients who underwent routine optometric and ophthalmological examinations at the Eye Rehabilitation and Vision Correction Center of Dr. Solomatina Ltd. in Riga, Latvia. The study protocol was approved by the Ethics Committee on Optometry and Vision Science Research of the Faculty of Exact Sciences and Technology of the University of Latvia (Committee Opinion No. 19-121/19). All procedures complied with the ethical standards outlined in the Declaration of Helsinki for research involving human subjects. Only de-identified data were used in the analysis, and no direct patient involvement occurred.

### Subjects

Participants were selected based on the following conditions: (1) Presence of a confirmed diagnosis of KC in both eyes for the KC group, determined through clinical evaluation and corneal tomography. (2) Presence of regular astigmatism, including with-the-rule (WTR), against-the-rule (ATR) or oblique astigmatism (minimum of 0.50 diopters (D) and maximum of 6.00 D of corneal astigmatism), without clinical signs of KC. (3) No clinical signs or diagnostic criteria of KC based on a standard screening protocol. (4) No history of ocular surgery or corneal cross-linking prior to the date of examination. Patient records were excluded under the following conditions: (1) Incomplete or missing data in key parameters, such as epithelial thickness profile, topography, or corrected visual acuity. (2) Signs of pellucid marginal degeneration, post-LASIK ectasia, or irregular astigmatism not attributable to KC. (3) Patients who had worn soft contact lenses within 10 days prior to the ophthalmologic examination were excluded from the study. (4) Prior use of rigid gas-permeable or scleral contact lenses within three weeks prior to examination. (5) Presence of other ocular pathologies affecting the cornea or retina (e.g., infections, dystrophies, trauma). (6) Systemic diseases with known corneal manifestations (e.g., diabetes mellitus, connective tissue disorders). Regular astigmatism was selected as the control condition because it reflects a non-ectatic, anatomically stable corneal shape variation and provides a clinically realistic comparator for distinguishing keratoconus-related epithelial remodeling from physiological epithelial changes associated with corneal toricity [23].

### Measurements

All measurements were extracted from clinical records and conducted by trained ophthalmologists and optometrists. Refractive error, including sphere, cylinder, and axis, was determined using subjective refraction with a trial frame and trial lenses, refined according to patient responses. This process was guided by the baseline values obtained through objective autorefraction using the Topcon KR-800 autorefractor (Topcon Corporation, Tokyo, Japan). The following tomographic and pachymetric parameters were obtained using the Pentacam HR (Oculus Optikgeräte GmbH, Wetzlar, Germany), a high-resolution rotating Scheimpflug imaging system: central corneal thickness (CCT) in microns (µm), thinnest corneal thickness (TCT) in µm, Steep keratometry (D), and total anterior corneal astigmatism (D), calculated as the vectorial difference between flat and steep keratometric readings. These measurements were acquired under non-contact conditions and automatically centered on the corneal apex using the Pentacam’s internal fixation system.

**Fig. 1:**
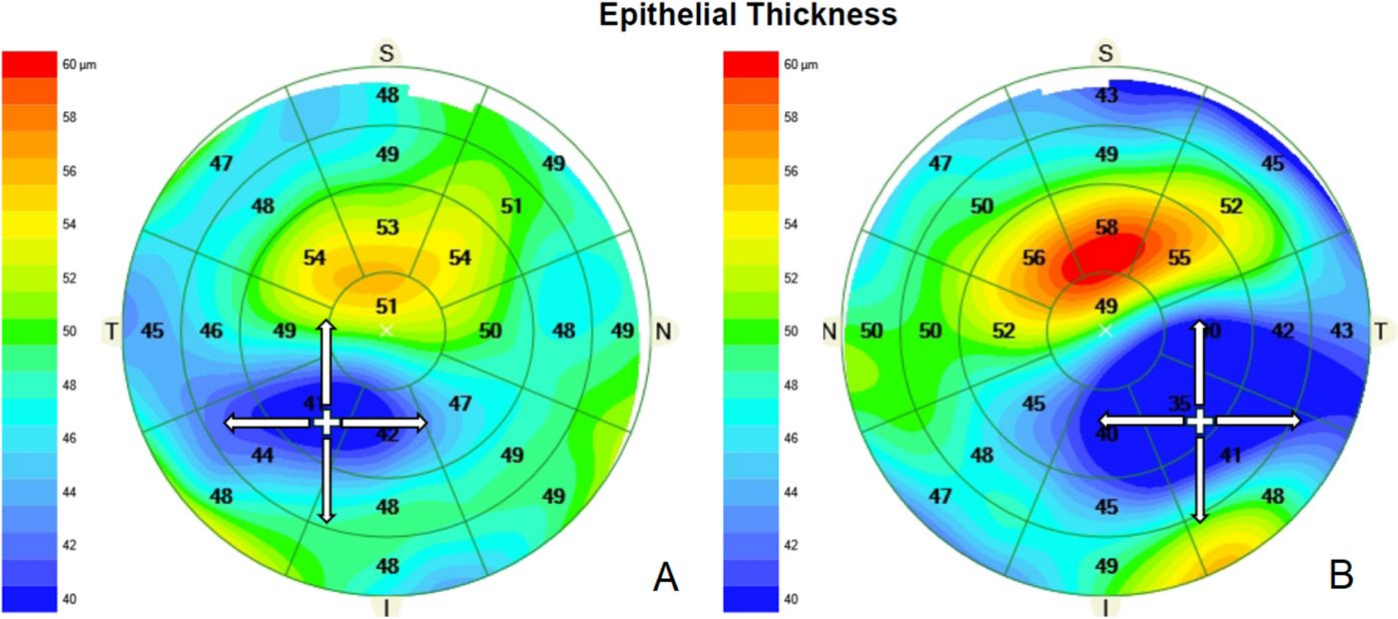
Epithelial thickness maps in keratoconus stage IA (A, right eye) and stage IB (B, left eye). The epithelial thickness gradient is more pronounced in stage IB, with marked thinning over the cone and peripheral thickening, reflecting advanced epithelial remodeling compared to stage IA

Quantitative analysis of the corneal epithelial thickness profile was performed using the Cirrus HD-OCT 5000 (Carl Zeiss Meditec AG, Jena, Germany). This device utilizes spectral-domain optical coherence tomography with high axial resolution, enabling detailed visualization of the epithelial layer across the central 9—mm zone. In each case, epithelial thickness was recorded at: the cone apex (see below for localization method), and four peripheral points located 3 mm away from the apex in the superior, temporal, nasal, and inferior directions (Fig. 1). ∆ET —defined as the difference between peripheral and central ET— was calculated for each meridian (∆ET = Peripheral ET − Apex ET).

In eyes with KC, the location of the ectatic apex was identified using axial curvature maps obtained with the Atlas 9000 corneal topographer (Carl Zeiss Meditec AG, Dublin CA, USA). The point of maximum anterior corneal steepening was manually localized and its position recorded in a Cartesian coordinate system relative to the geometric center of the cornea (horizontal [x] and vertical [y] displacement in millimeters). These relative coordinates were subsequently transferred to the ET maps acquired with the Cirrus HD-OCT, using the corneal center as a common anatomical reference to ensure spatial correspondence between devices (Fig. 1). In the regular astigmatism group, the same relative coordinates derived from the KC cohort were applied to the epithelial maps to allow homologous spatial sampling and direct comparison of ET gradients, rather than to imply the presence of an ectatic apex. All scans were acquired under fixation with automatic centration enabled in each device. Coordinate transfer followed a standardized protocol using the geometric corneal center as a shared reference and a consistent Cartesian convention (x horizontal, y vertical). Peripheral sampling points were defined at a fixed 3—mm radial distance from the apex along the four cardinal meridians to ensure reproducible spatial positioning. Keratoconus severity was assessed using the ABCD grading system provided by the Pentacam (Oculus GmbH, Wetzlar, Germany), based on anterior (A) curvature within a 3.0 mm zone [11]. Early-stage keratoconus (Stage I) was classified according to the Amsler–Krumeich [2] system and further subdivided based on steep keratometry into subclassification A (Steep K < 46 D) and subclassification B (46 D ≤ Steep K < 48 D) to capture severity differences within early disease.

### Statistical analysis

The Kolmogorov–Smirnov test was performed to assess the normality of the data distribution. For non-normally distributed data, independent Mann–Whitney U tests were used to compare means. Descriptive statistics, including mean, standard deviation (SD), and range, were presented for all variables. Correlation analyses between ∆ET values and keratoconus severity indices were performed using Spearman’s rank correlation coefficient, given the non-parametric distribution of the data. Statistical significance was set at a *p* < 0.05. The sample size was calculated using a two-sided independent samples t-test to detect a mean difference in ET between KC and control eyes. Although the sample size calculation was based on a two-sided independent samples t-test, this approach provides a valid planning approximation. Due to non-normal data distribution, the Mann–Whitney U test was used for the final analyses. Based on Dutra et al. [24], we assumed a mean ET of 49.19 ± 3.50 µm in controls and 44.65 ± 5.97 µm in KC eyes. Setting the significance level (α) at 0.05 and statistical power at 80% (1–β = 0.80), the required sample size was estimated at 19 eyes per group (38 eyes in total).

In the KC group, both eyes of each participant were included for analysis. This decision was based on previous evidence indicating that eyes affected by keratoconus may exhibit significant asymmetry and behave as independent units [25–27], particularly in early or asymmetric stages [28]. Therefore, treating both eyes as independent observations is considered acceptable and informative in such populations. In contrast, for the control group—where inter-eye correlation is expected to be stronger—only one eye per subject was randomly selected to avoid statistical bias due to paired data.

To assess the diagnostic performance of ∆ET parameters in distinguishing KC from regular astigmatism, receiver operating characteristic (ROC) curve analysis was performed. The area under the ROC curve (AUC) was calculated to determine the discriminative ability of each gradient variable (i.e., superior, inferior, nasal, and temporal ET differences from the center, as well as their mean). The optimal cut-off point for each parameter was identified using the Youden index (sensitivity + specificity – 1), which maximizes the combined sensitivity and specificity. Sensitivity, specificity, and AUC values were reported with 95% confidence intervals. AUC values between 0.7 and 0.9 were considered to indicate acceptable to excellent discriminatory ability [29–31]. To address potential inter-eye dependency, an additional sensitivity analysis was performed including one randomly selected eye per subject in both groups. ROC analysis was repeated in this reduced dataset.

## Results

A total of 396 eyes from 199 patients were included in the study. The KC group comprised 208 eyes from 104 patients (79 men and 25 women), with a mean age of 34.02 ± 10.00 years (range: 15–67). The regular astigmatism group included 188 eyes from 95 patients, with a mean age of 33.70 ± 6.15 years (range: 20–54). Among these, 62 were men and 33 were women. Table 1 summarizes the refractive and corneal parameters of the KC and regular astigmatism groups. Statistically significant differences were observed between groups in most refractive values, including sphere, cylinder, spherical equivalent (SE), and the best corrected visual acuity (CDVA). Likewise, tomographic measurements such as CCT and TCT, as well as steep keratometry values, were significantly lower or steeper in the KC group compared to the control group. Axial orientation also showed a distinct distribution between the two cohorts.

**Table 1:** Descriptive statistics of ocular and refractive parameters in keratoconus and regular astigmatism groups

Variable^a^	Keratoconus (*n* = 208)	Regular astigmatism (*n* = 188)	*p*-value
Age (years)	34.02 ± 10.00 (15–67)	33.70 ± 6.15 (20–54)	.344
Sphere (D)	− 1.27 ± 2.66 (− 20.00–2.75)	− 2.13 ± 2.18 (− 8.25–1.50)	< .001*
Cylinder (D)	− 1.58 ± 1.40 (− 6.00–0.00)	− 2.45 ± 0.91 (− 5.75–-0.25)	< .001*
Axis (°)	77.29 ± 55.68 (0–175)	98.57 ± 72.41 (0–180)	< .001*
SE (D)	− 2.04 ± 2.75 (− 21.25–1.25)	− 3.35 ± 2.16 (− 9.75–0.25)	< .001*
CDVA (logMAR)	0.11 ± 0.21 (− 0.20–1.00)	0.005 ± 0.096 (− 0.20–1.00)	< .001*
CCT (µm)	465.26 ± 45.61 (267–607)	530.43 ± 43.68 (456–597)	< .001*
TCT (µm)	471.06 ± 50.16 (112–610)	535.21 ± 24.61 (476–602)	< .001*
Corneal Astigmatism (D)	2.88 ± 3.30 (0.00–4.70)	2.34 ± 1.03 (0.50–5.20)	*.309*
Steep Keratometry (D)	46.80 ± 4.09 (40.53–71.84)	43.98 ± 1.71 (40.52–48.10)	< .001*

D, diopters; SE, spherical equivalent; CDVA, corrected distance visual acuity; logMAR, logarithm of the minimum angle of resolution; CCT, central corneal thickness; TCT, thinnest corneal thickness

^a^Expressed in mean ± standard deviation (minimum, maximum)

^*^Statistically significant level with U of Mann Whitney test for independent data

In the KC group, the apex of the cone was typically located slightly below the corneal center, around (x = –0.42 ± 0.85 mm, y = –0.94 ± 0.60 mm) in right eyes and (x = 0.52 ± 0.80 mm, y = –1.05 ± 0.62 mm) in left eyes; this location was used as the reference point for ET measurements in the regular astigmatism group. Table 2 summarizes the differences in ET between the KC and regular astigmatism groups. Statistically significant thinning of the central epithelium was observed in eyes with KC compared to those with regular astigmatism (*p* < 0.001). Although the temporal and nasal regions did not show statistically significant differences, the superior region was slightly thinner in the KC group (*p* = 0.013), while the inferior region showed no differences between groups. Of relevance, the ΔET were markedly steeper in the KC group for all quadrants: temporal (ΔET-T), superior (ΔET-S), nasal (ΔET-N), and inferior (ΔET-I). These gradients were all significantly greater in the KC group compared to the regular astigmatism group (*p* < 0.001), highlighting the characteristic epithelial remodeling pattern associated with ectatic disease.

**Table 2:** Comparison of epithelial thickness measurements between keratoconus and regular astigmatism groups

Epithelial thickness (µm)^a^	Keratoconus (*n* = 208)	Regular astigmatism (*n* = 188)	*p*-value
Central	38.55 ± 5.42 (25–55)	48.78 ± 3.94 (41–64)	< .001 *
Temporal	45.75 ± 4.41 (36–60)	42.21 ± 15.28 (36–83)	.318
Superior	47.65 ± 4.49 (37–60)	46.41 ± 3.82 (34–59)	.013 *
Nasal	46.94 ± 4.44 (33–59)	42.42 ± 14.77 (39–72)	.179
Inferior	47.60 ± 5.00 (30–62)	47.63 ± 3.94 (36–59)	.913
∆ Temporal – Central	7.20 ± 5.15 (− 6–25)	− 1.63 ± 4.35 (− 6–19)	< .001 *
∆ Superior – Central	9.10 ± 5.81 (− 5–30)	− 2.13 ± 2.35 (− 10–7)	< .001 *
∆ Nasal – Central	8.39 ± 5.44 (− 5–33)	− 1.43 ± 2.02 (− 8–8)	< .001 *
∆ Inferior – Central	9.05 ± 5.97 (− 6–37)	− 1.03 ± 1.85 (− 8–4)	< .001 *

^a^Expressed in mean ± standard deviation (minimum, maximum)

^*^Statistically significant level with U of Mann Whitney test for independent data

Table 3 presents the ET profiles in Stage I KC eyes subdivided by two classification criteria. In the Steep K-based groups, subclassification B (more advanced, 48 D > Steep K ≥ 46 D) showed significantly thinner central epithelium and steeper ΔET in all quadrants compared to subclassification A (early stage, 46 D > Steep K), with p < 0.05 in all cases except for peripheral values. In the TCT-based groups, subclassification B (450–470 µm) showed slightly higher ΔET values, with significant differences in the nasal gradient (p = 0.028) and a trend in the temporal quadrant (p = 0.045), although central ET did not differ significantly between B and A subclassification.

**Table 3:** Subclassification of Stage 1 keratoconus based on steep keratometry

Epithelial thickness (µm)^a^	Stage I *Subclassification A*^*1*^ *46 D* > *Steep K* (*n* = 105)	Stage I *Subclassification B*^*1*^ *48 D* > *Steep K* ≥ *46 D* (*n* = 44)	*p*-value
Central	40.54 ± 5.55 (25–55)	38.52 ± 4.82 (30–55)	.024*
Temporal	46.13 ± 4.24 (36–58)	45.75 ± 4.00 (37–55)	.688
Superior	47.75 ± 4.15 (39–58)	48.39 ± 4.61 (41–60)	.495
Nasal	47.01 ± 4.34 (39–58)	47.50 ± 3.05 (40–55)	.319
Inferior	47.70 ± 5.34 (30–62)	47.64 ± 4.34 (33–56)	.889
∆ Temporal—Central	5.59 ± 4.85 (− 6–25)	7.23 ± 5.11 (− 5–19)	.029*
∆ Superior—Central	7.21 ± 5.17 (− 2–30)	9.86 ± 6.82 (− 5–24)	.009*
∆ Nasal—Central	6.47 ± 5.13 (− 5–33)	8.98 ± 4.92 (− 5–21)	< .001*
∆ Inferior—Central	7.15 ± 5.60 (− 4–37)	9.11 ± 5.95 (− 6–21)	.020*

^1^Based on stage I Steep K and refraction range definition of Amsler-Krumeich classification

^a^Expressed in mean ± standard deviation (minimum, maximum)

^*^Statistically significant level with U of Mann Whitney test for independent data

To further characterize epithelial remodeling, mean ΔET was defined as the average of the four peripheral–central ET differences (temporal, superior, nasal, and inferior). This parameter reflects the overall steepness of the epithelial profile. The mean ΔET was significantly higher in the KC group (8.43 ± 4.97 µm) compared to the regular astigmatism group (–1.55 ± 1.77 µm; p < 0.001). Within the KC group, the gradient increased with disease severity: 6.60 ± 4.67 µm in stage IA, 8.79 ± 5.08 µm in stage IB, and 11.42 ± 3.84 µm in moderate-to-severe stages (stages II–IV), with statistically significant differences (*p* < 0.001). Spearman correlation analysis revealed a moderate positive correlation between mean ΔET and Steep K (ρ = 0.521, *p* < 0.001), and a moderate negative correlation with TCT (ρ = –0.641, *p* < 0.001), indicating that greater ΔET are associated with steeper keratometry values and thinner corneas.

**Fig. 2:**
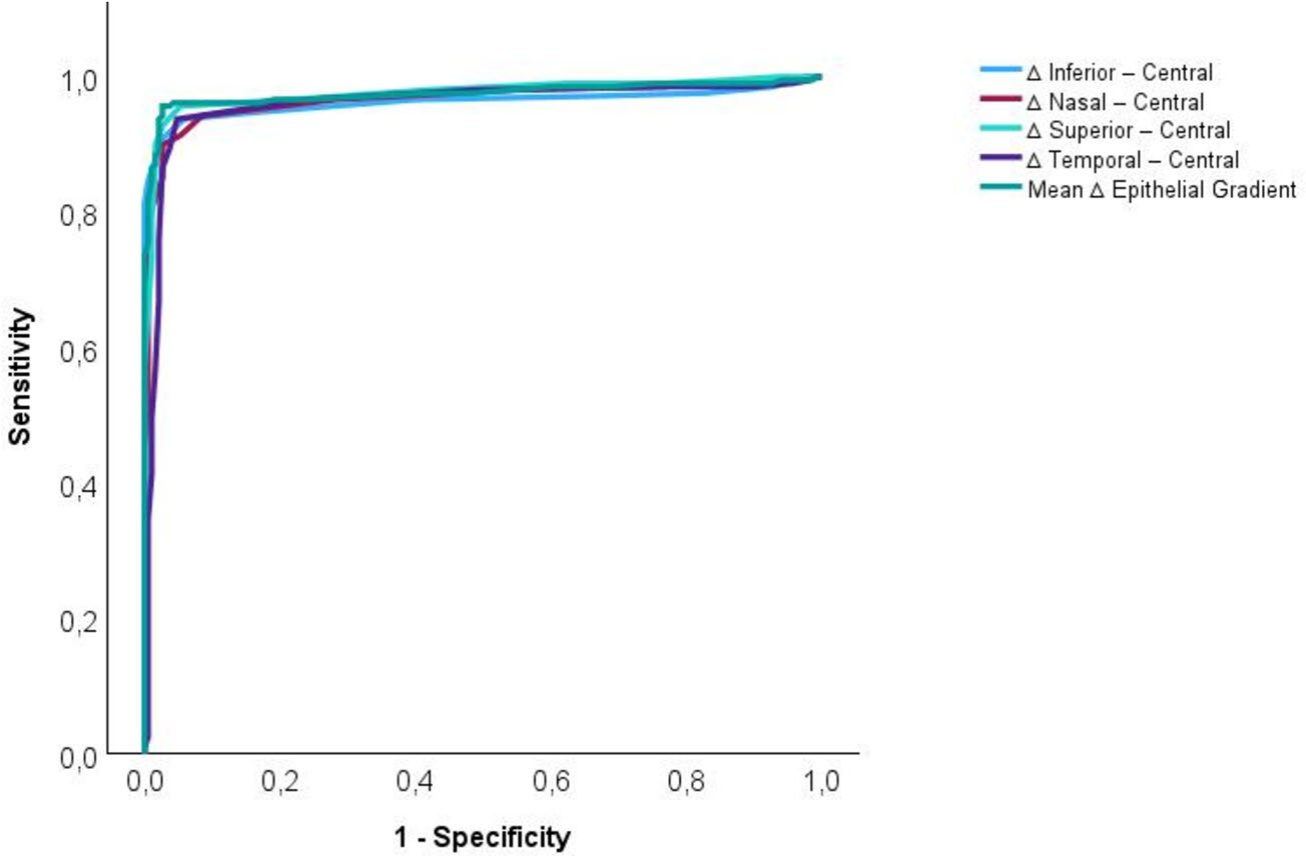
Receiver operating characteristic (ROC) curves for epithelial thickness gradient parameters in the detection of keratoconus. Each curve represents the diagnostic performance of the difference in epithelial thickness between the peripheral and central cornea at four anatomical locations (inferior, nasal, superior, and temporal), as well as the mean epithelial thickness gradient calculated from all four positions. The x-axis represents—specificity (false positive rate), and the y-axis represents sensitivity (true positive rate)

ROC curve analysis was conducted to evaluate the diagnostic performance of ∆ET parameters in distinguishing keratoconus from regular astigmatism (Fig. 2). All variables demonstrated excellent discriminatory ability, with AUC values ranging from 0.960 to 0.976. The mean ∆ET yielded an AUC of 0.975 (95% CI: 0.958–0.992), with an optimal cut-off point of 1.125 µm, corresponding to a sensitivity of 95.7% (95% CI: 92.0–97.7) and a specificity of 97.3% (95% CI: 93.8–98.9). When restricting the analysis to one eye per subject, the diagnostic performance remained unchanged (AUC = 0.979; 95% CI: 0.964–0.995), confirming the robustness of the findings. Among the peripheral gradients, the superior location showed the highest AUC (0.976), followed by nasal (0.967), inferior (0.963), and temporal (0.960) (Table 4). These findings support the potential utility of ∆ET metrics as non-invasive diagnostic biomarkers for KC.

**Table 4:** Summary of ROC curve analysis for epithelial thickness gradient variables. The table reports the area under the curve (AUC), 95% confidence intervals, optimal cut-off points (based on the Youden index), sensitivity, and specificity for each parameter

Variable	AUC	95% CI Lower	95% CI Upper	Optimal Cut-off*	Sensitivity	Specificity
∆ Temporal—Central	0.960	0.938	0.983	0.500	0.938	0.952
∆ Superior—Central	0.976	0.961	0.991	0.500	0.957	0.947
∆ Nasal—Central	0.967	0.948	0.986	2.500	0.899	0.973
∆ Inferior—Central	0.963	0.941	0.985	2.500	0.909	0.973
Mean ∆ET	0.975	0.958	0.992	1.125	0.957	0.973

AUC, area under the curve; CI, confidence interval; ET, epithelial thickness

^*^Cut-off values were selected to maximize the sum of sensitivity and specificity (Youden’s J index) and may vary slightly depending on the distribution of values in the sample

The mean ∆ET demonstrated excellent diagnostic performance in the early stages of KC. When analyzed by stage, the area under the ROC curve was 0.965 (95% CI: 0.937–0.994) for Stage IA and 0.955 (95% CI: 0.902– 1.009) for Stage IB. These values indicate outstanding discriminatory power in both subgroups. The optimal threshold for detecting keratoconus in Stage IA was 1.125 µm, yielding a sensitivity of 93.6% (95% CI: 86.8– 97.0) and a specificity of 97.4% (95% CI: 93.6 – 99.0). For Stage IB, the most accurate cut-off was 1.250 µm, achieving a sensitivity of 92.9% (95% CI: 81.0– 97.5) and a specificity of 96.3% (95% CI: 81.7– 99.3), based on the maximum Youden index. These findings highlight that even in the earliest detectable forms of keratoconus, the mean ∆ET serves as a sensitive and specific diagnostic biomarker. The progressive increase in the threshold across stages also reflects its potential utility in both early detection and staging of the disease, supporting the integration of epithelial mapping into routine keratoconus screening protocols.

## Discussion

The present study confirms that ΔET can serve as a quantifiable biomarker for early detection assessment in KC. In our cohort, ΔET values were significantly steeper in KC eyes than in eyes with regular astigmatism, and this steepening showed a clear correlation with disease severity. ΔET complements existing epithelial indices by reflecting localized, apex-centered epithelial remodeling rather than absolute thickness or global variability.

### Doughnut pattern and compensatory thickening

Our results confirm the presence of the characteristic “doughnut pattern” of epithelial remodeling in KC, marked by localized central thinning surrounded by a peripheral thickening ring. This pattern has been consistently described, most notably by Reinstein et al.[12, 13], who used very high-frequency digital ultrasound to show that epithelial thinning directly overlays the stromal cone, while the surrounding annulus thickens to mask irregularities and preserve the anterior curvature. Using Scheimpflug imaging before and after epithelial debridement, Franco et al.[21] further confirmed this architecture, documenting a central thinning down to ~ 20 μm and peripheral thickening to ~ 40 μm. These changes intensified with disease severity, aligning with our findings of increasingly steep ΔET from Stage I onward.

### Epithelial thickness gradients in early keratoconus

One of the key insights from our study is the demonstration that ΔET are already present and significantly altered in the earliest stages of KC, even when other topographic indices may still appear within normal limits [24, 32, 33]. This supports the notion that epithelial remodeling may precede or at least parallel structural stromal changes, as proposed by several authors. Naujokaitis et al. provided compelling evidence that localized ET differences—especially in the inferior-temporal region—are elevated not only in tomographically evident KC but also in the clinically unaffected fellow eyes of patients with asymmetric disease [34]. Their reported inter-zonal differences support our approach of using peripheral-to-central ΔET as a surrogate for early remodeling, potentially even before biomechanical decompensation becomes evident.

Similarly, Temstet et al. identified subtle epithelial thinning in the thinnest corneal zone of form fruste keratoconus (FFKC) cases, undetectable by topography or Scheimpflug parameters alone [35]. This finding parallels our observation that epithelial steepening can unmask early ectatic behavior. Further support comes from Zhou et al., who mapped epithelial and stromal thickness across steep and flat meridians and found that inferotemporal thinning of the epithelium—with corresponding supranasal thickening—was a distinct marker even in eyes with moderate astigmatism [23].

### Correlation of ΔET with severity indices

Our findings reveal a consistent and progressive increase in ΔET with advancing KC severity. This gradation is consistent with prior evidence indicating that epithelial behavior reflects the underlying biomechanical and morphological changes that occur as KC advances. In the study by Franco et al., ET decreased significantly from Stage I (23 μm) to Stage III (18 μm), while peripheral thickening became more pronounced [21]. This progression was accompanied by steeper central keratometry and increased variability in ET maps (as measured by standard deviation), aligning with our observation that ΔET becomes steeper in more advanced subgroups—even within Stage I. Koh et al. further highlighted how epithelial and stromal thinning worsens with clinical severity [18]. Importantly, their analysis in a large cohort using swept-source OCT supports the notion that epithelium is not only reactive but also closely tracks disease evolution. This correlation is also statistically reinforced in the neural network models presented by Silverman et al., who demonstrated that parameters derived from epithelial and stromal thickness maps accurately predicted keratoconus severity [17].

### ΔET as a screening tool and future perspectives

The diagnostic potential of ΔET lies in staging KC and in detecting epithelial remodeling patterns that may be present even when topographic or tomographic alterations are subtle or not evident. Toprak et al. reported distinct epithelial distribution patterns even in FFKC and subclinical KC. Although the optimal ΔET cut-offs were numerically small, they reflect a shift in epithelial gradient behavior rather than large absolute thickness differences. Nevertheless, given that epithelial mapping variability may be in the micrometer range, device-specific validation is required before applying fixed thresholds in routine screening. Their use of epithelium-to-stroma (E/S) ratios and peripheral epithelial thinning, particularly in the superior-nasal sectors, suggests that ΔET-based models could enhance the early detection of ectatic disease when integrated into a multivariable approach [33]. Similarly, Silverman et al. showed that combining ET data with Scheimpflug metrics significantly improved classifier performance, achieving near-perfect sensitivity and specificity for established cases, and setting the stage for its use in subclinical detection [36]. The potential for ΔET to serve as a screening parameter is supported by its non-invasiveness and the increasing availability of epithelial mapping in clinical OCT platforms; however, repeatability and device-specific thresholds must be considered. However, as highlighted by Wang et al., measurement variability increases with peripheralization and disease severity, and discrepancies between devices (e.g., RTVue vs MS-39) must be considered when standardizing ΔET cutoffs across clinical settings [37]. Emerging evidence suggests that the ocular surface microbiome may contribute to keratoconus progression, as advanced stages show distinct microbial profiles associated with local immune dysregulation and potential epithelial dysfunction [38].

### Limitations

This study’s cross-sectional design precludes conclusions regarding longitudinal change or progression detection; therefore, any monitoring implications remain hypothetical until validated in prospective longitudinal cohorts. Future prospective and longitudinal studies are warranted to evaluate the role of ΔET in early screening, disease monitoring, and integration with existing tomographic and biomechanical indices. Cone apex localization using axial curvature may vary in irregular cases, affecting ΔET precision. Device-related differences also limit comparison across imaging systems. ΔET thresholds are likely device-specific, and their clinical implementation will require platform-dependent normative databases and independent validation across imaging systems. Measurements were confined to 3 mm from the apex, possibly missing peripheral remodeling. Lastly, staging based on Amsler-Krumeich may lack posterior surface data; future work should consider the ABCD system. Although regular astigmatic controls were used, alternative control groups—such as healthy subjects without astigmatism or patients with irregular astigmatism without ectasia—could have been included, an aspect that should be addressed in future prospective studies.

A direct comparison between ΔET and established tomographic or biomechanical indices (e.g., BAD-D, CBI, TBI) was not feasible, as tomographic and biomechanical data were not available for all subjects. Consequently, the incremental diagnostic value of ΔET beyond established multimodal screening parameters cannot be determined from the present study. ΔET should therefore be interpreted as a complementary epithelial-based metric rather than a replacement for existing tomographic or biomechanical indices. Future studies integrating epithelial thickness mapping with tomography and corneal biomechanics are necessary to assess whether ΔET provides additive discriminatory value, particularly in early or subclinical keratoconus.

Although regular astigmatic corneas were selected as controls to provide a clinically relevant comparison group, the absence of a cohort of healthy subjects with minimal or no astigmatism limits the ability to fully characterize ΔET behavior in physiologically normal corneas. Regular astigmatism itself may involve mild epithelial compensation along the principal meridians, potentially influencing epithelial gradient patterns. Therefore, the extent to which ΔET differs between normal low-astigmatism corneas and regular astigmatism remains to be clarified. Future studies should include both emmetropic or low-astigmatism healthy controls and additional non-ectatic irregular corneal conditions to better define the specificity of ΔET for keratoconus-related epithelial remodeling.

Cone apex localization and manual coordinate transfer between devices may introduce minor spatial misalignment. Because ΔET is calculated at a fixed 3—mm distance from the apex, small localization errors could influence gradient magnitude. Future studies should assess inter-operator variability or incorporate automated image registration to improve spatial reproducibility. Repeatability and reproducibility of Cirrus HD-OCT epithelial mapping were not formally assessed in this dataset, and future studies should establish device-specific repeatability limits before clinical implementation of fixed ΔET thresholds.

## Conclusion

The ΔET offers a reliable, quantifiable indicator of early corneal remodeling in KC. This study demonstrates that ΔET is significantly steeper in KC compared to regular astigmatism and increases with disease severity, even within Stage I subgroups. The moderate correlations between mean ΔET, Steep K, and TCT support its role as a sensitive, non-invasive diagnostic biomarker for early-stage KC. ΔET complements existing epithelial indices by reflecting localized, apex-centered epithelial remodeling rather than absolute thickness or global variability. ROC curve analysis confirmed the excellent diagnostic performance of ΔET parameters, with AUC values up to 0.976 and high sensitivity and specificity, even in Stage I subgroups. Integrating ΔET into clinical evaluation may enhance early detection and classification of KC. Longitudinal studies are required to determine whether ΔET is suitable for monitoring progression over time.

## Data Availability

The data generated and analyzed during this study are not publicly available due to copyright restrictions. However, the data can be requested from the corresponding author under reasonable conditions and for academic or research purposes.

## Declarations

### AI use declaration

Artificial intelligence tools were used solely to assist with grammar correction, technical phrasing, and writing clarity during the preparation of this manuscript.
